# Uncovering the Role of Natural and Synthetic Small Molecules in Counteracting the Burden of α-Synuclein Aggregates and Related Toxicity in Different Models of Parkinson’s Disease

**DOI:** 10.3390/ijms241713370

**Published:** 2023-08-29

**Authors:** Salihu Mohammed, Isabella Russo, Ileana Ramazzina

**Affiliations:** 1 Department of Medicine and Surgery, University of Parma, Via Gramsci 14, 43126 Parma, Italy; salihu.mohammed@unipr.it; 2 Department of Molecular and Translational Medicine, University of Brescia, Via Europa 11, 25123 Brescia, Italy; isabella.russo@unibs.it; 3 IRCCS Istituto Centro San Giovanni di Dio Fatebenefratelli, Via Pilastroni 4, 25125 Brescia, Italy; 4 Centre for Molecular and Translational Oncology (COMT), University of Parma, Parco Area delle Scienze 11/a, 43124 Parma, Italy; 5 Biostructures and Biosystems National Institute (INBB), Viale Medaglie d’Oro 305, 00136 Rome, Italy

**Keywords:** proteostasis, molecular chaperones, α-Synuclein, small molecules, Parkinson’s disease

## Abstract

A proteostasis network represents a sophisticated cellular system that controls the whole process which leads to properly folded functional proteins. The imbalance of proteostasis determines a quantitative increase in misfolded proteins prone to aggregation and elicits the onset of different diseases. Among these, Parkinson’s Disease (PD) is a progressive brain disorder characterized by motor and non-motor signs. In PD pathogenesis, alpha-Synuclein (α-Syn) loses its native structure, triggering a polymerization cascade that leads to the formation of toxic inclusions, the PD hallmark. Because molecular chaperones represent a “cellular arsenal” to counteract protein misfolding and aggregation, the modulation of their expression represents a compelling PD therapeutic strategy. This review will discuss evidence concerning the effects of natural and synthetic small molecules in counteracting α-Syn aggregation process and related toxicity, in different in vitro and in vivo PD models. Firstly, the role of small molecules that modulate the function(s) of chaperones will be highlighted. Then, attention will be paid to small molecules that interfere with different steps of the protein-aggregation process. This overview would stimulate in-depth research on already-known small molecules or the development of new ones, with the aim of developing drugs that are able to modify the progression of the disease.

## 1. Introduction

The proteostasis network (PN) constitutes the set of cellular components involved in establishing and maintaining proteostasis, also called cellular protein homeostasis. The main tasks of PN are protein synthesis, folding, localization, and degradation; all these branches work in a coordinated manner to ensure cellular proteome integrity. During the protein folding process, a nascent polypeptide chain acquires its three-dimensional conformation and becomes a biologically functional protein. The main players of the protein folding branch are the chaperone systems, the folding enzymes, and the machinery of the endoplasmic reticulum (ER) [1]. Indeed, in healthy conditions, cells can obtain correctly folded proteins, but also refold the misfolded ones or deliver them to degradation systems. When the balance between the folding and unfolding proteins fails, due to the biological processes of aging or stress conditions, misfolded proteins overwhelm the control systems and trigger the formation of toxic aggregates. The latter impair cellular functions, and their accumulation with time leads to the onset and progression of protein-misfolding diseases, such as Alzheimer’s disease (AD) and Parkinson’s disease (PD) [2,3,4].

The biosynthesis of proteins, their post-translational modification, and folding take place predominantly in the ER, thus this cellular organelle plays a crucial role in proteostasis. Unfolded proteins are recognized by the ER-associated degradation (ERAD) pathway and are retrotranslocated from the lumen or membrane of the ER into the cytoplasm, where they are degraded by the proteasome. The increasing burden of unfolding proteins causes ER stress. To counteract the formation of misfolded protein aggregates and in an attempt to maintain protein homeostasis, cells trigger the Unfolded Protein Response (UPR), both in the ER and mitochondria. The UPR mechanism allows (i) to increase the expression of molecular chaperones which aid the protein folding process; (ii) to reduce the burden of misfolded proteins through ERAD and lysosome degradation pathways; and (iii) to reduce the expression of proteins that demand the assistance of molecular chaperones to achieve the functional shape [3,5].

Molecular chaperones, and their related co-chaperones, work in all the branches of PN and in different cell compartments (i.e., ER, mitochondria, and cytoplasm). They help in the maintenance of cellular proteostasis through their involvement in the folding of novel proteins, inhibition, and reversal of misfolded and aggregated proteins, and directing unrepairable misfolded proteins to the degradation systems [6]. Interestingly, also in the extracellular milieu, molecular chaperones can stabilize proteins with incorrect structure and maintain them in a soluble state, thus inhibiting aggregation events, and facilitating their efficient clearance through receptor-mediated endocytosis [7,8]. Molecular chaperones were first identified as Heat Shock Proteins (HSPs) due to their increased expression in response to a heat shock treatment. Nowadays, it is well-recognized that the upregulation of molecular chaperones is triggered by different stressor stimuli (e.g., chemicals and heavy metals, hypoxia, and changes in physiological pH) [6,9]. The most reported classification of molecular chaperones is in reference to their molecular weight; on this evidence, they are classified into five classes: HSP60, HSP70, HSP90, HSP104, and the small HSPs [10].

As mentioned above, many neurodegenerative pathologies are associated with PN impairments. Among these, AD and PD are the two major neurodegenerative illnesses worldwide [11]. PD is an age-related neurodegenerative disorder characterized by cardinal signs, namely tremors, bradykinesia, rigidity, and postural instability. Literature data also highlight the presence of non-motor symptoms (e.g., low blood pressure upon standing, excessive sweating, urinary incontinence, constipation, impaired stomach emptying, and neuropsychiatric disturbances) that can precede cardinal motor features by years [12,13]. Both environmental and genetic factors are related to the onset of PD; most cases are sporadic, and only 5% are of genetic origin [14,15].

In PD, the failure of PN leads to the accumulation of intracellular proteinaceous aggregates called Lewy Bodies (LBs), which are composed mainly of alpha-Synuclein (α-Syn) and represent the hallmark of the disease. During PD progression, α-Syn oligomers spread from neuron to neuron and trigger the release of inflammatory mediators and the induction of neuroinflammatory processes [16]. α-Syn aggregates are commonly located in the Central Nervous System (CNS). However, they have also been described in other areas of the peripheral nervous system, such as the enteric neurons. This finding supports the hypothesis of the bidirectional communication between the enteric and central nervous systems (gut–brain axis) [17].

α-Syn is expressed in different parts of the brain, and different functions are associated with its physiological forms, such as the regulation of dopamine synthesis, the dopamine vesicle dimension, and the localization of dopamine transporters [18]. The mechanics by which α-Syn loses its physiological structure and gains a toxic form (i.e., misfolded monomers, oligomers, and fibrils) are not fully elucidated, while there are no doubts that its “loss-and-gain” structure plays a role in the pathogenesis of PD.

The therapeutic interventions to manage PD aim to improve the cardinal and non-motor symptoms, through the administration of drugs (e.g., levodopa, dopamine agonists, rehabilitation programs), and more recently, by the use of device-aided therapies (e.g., continuous subcutaneous apomorphine infusion, or continuous jejunal infusion of L-dopa-carbidopa intestinal gel and deep brain stimulation) [19,20]. However, these approaches are not able to modify the cause of the disease. Since an increasing body of literature highlights the pivotal role of α-Syn in the progression and spreading of the pathology, it becomes necessary to develop strategies that can counteract the aggregation process of α-Syn and the related detrimental effects. In this regard, different approaches have been proposed, aiming to reduce α-Syn production, hamper the aggregation pathway and the cell-to-cell uptake of α-Syn aggregates, and increase their clearance [21,22,23,24,25]. Accordingly, there is an increasing body of literature that explores the role of molecular chaperones in counteracting the multistep aggregation pathway of α-Syn and related toxicity. It has been demonstrated that different HSPs prevent α-Syn aggregation in vitro, thanks to their ability to bind to different α-Syn regions. Interestingly, the results highlight that different molecular chaperones (e.g., ubiquitous HSPs, holdases, and members with disaggregation activity) can interact alone and/or synergistically to counteract α-Syn aggregation [26]. Moreover, the overexpression of HSPs, such as HSP70 and HSP27, reduced the number of α-Syn aggregates as well as their associated cytotoxicity [27,28,29]. Interestingly, chaperones can also influence the function of other proteins involved in biochemical pathways essential to maintain healthy cells and they can play their roles in different intracellular compartments (e.g., cytoplasm, ER, mitochondria) and the extracellular milieu [7,30,31,32]. Moreover, a recent study highlights the possible use of a pool of chaperones as molecular biomarkers of PD [33]. Another explored therapeutic approach for PD is the use of chemical chaperones, namely small molecules that work in a “chaperone-like manner”. Precisely, chemical chaperones are defined as non-specific compounds that can stabilize different proteins; pharmacological chaperones instead bind to specific target protein(s). However, the common feature of these low-molecular-weight molecules is their ability to stabilize proteins and avoid their aggregation, performing a classical function associated with endogenous molecular chaperones, thus working in a “chaperone-like manner”. Moreover, most of these molecules offer stability and bioavailability, and do not require special storage conditions [34,35].

The idea of this review, taking into account the central role of the α-Syn aggregation process in PD pathogenesis, is to discuss the recent studies that investigate two of the main strategies extremely attractive for therapeutic interventions: (i) small molecules that affect molecular chaperones as a regulator of proteostasis, and (ii) small molecules that can interfere directly with the α-Syn aggregation events. Importantly, we analyse studies concerning both natural and synthetic compounds carried out in in vitro, cellular and in vivo models of PD, to provide an overview of these alternative approaches and to stimulate new research.

## 2. Natural and Synthetic Small Molecules That Affect Molecular Chaperones

**Figure 1:**
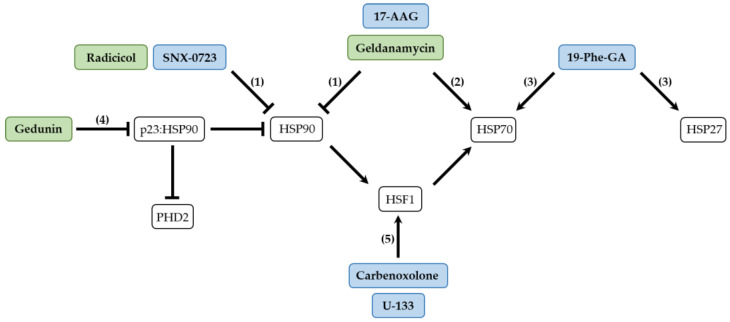
Schematic representation of the main molecular chaperones targeted by natural and synthetic small molecules that have been demonstrated in PD cellular and animal models. Geldanamycin, 17-AAG, SNX-0723 and Radicicol treatment (1) inhibit HSP90 expression/activity leading to the activation of HSF1, which then results in an increase in the expression of HSP70 and its activity. Geldanamycin and 17-AAG administration (2) directly result in an increase in the expression and activity of HSP70. 19-Phe-GA treatment (3) directly induces an increase in the expression and activity of HSP70 and HSP27. Gedunin treatment (4) disrupts the p23:HSP90 complex, leading to the proteasomal degradation of PHD2 and the inhibition of HSP90 activity; these events result in the activation of HSF1 and the increase in the expression of HSP70 and its activity. Carbenoxolone and U-133 administration (5) activate HSF1, which leads to HSP70 expression and activity. The increase in HSP70 expression and activity, as well as that of other chaperones like HSP27, lead to the reduction of α-Syn aggregates and related toxicity. Green box: natural small molecules; Blue box: derivative/synthetic small molecules. 17-AAG: 17-(Allylamino)-17-demethoxygeldanamycin; 19-Phe-GA: 19-phenyl-geldanamycin; PHD2: Prolyl Hydroxylase Domain Protein 2; HSP: Heat Shock Protein; HSF1: Heat Shock transcription Factor 1.

For about two to three decades now, many researchers have focused on small molecules that can target molecular chaperones in reducing PD neurotoxicity. The roles of molecular chaperones in the proper folding of native proteins or to counteract protein aggregation events are widely known. Thus, the discovery of molecules targeting chaperones is an ongoing research challenge to reduce the burden of toxic α-Syn aggregates in PD pathology and other synucleinopathies. In this section, the experimental achievements obtained by using natural and synthetic small molecules in cellular and animal models of PD will be discussed. In agreement, Figure 1 summarizes the interplay between the small molecules and their targeted molecular chaperones, and Table 1 highlights the main results reported in the articles included in this review.

It can be deduced from the literature data that HSP90 represents an attractive target to develop novel drugs against neurodegenerative diseases, as it is well known that HSP90 can influence the balance between α-Syn oligomeric and fibril forms with respect to ATP availability in cells. Furthermore, under unstressed conditions, HSP90 forms a complex with the Heat Shock transcription Factor 1 (HSF1), suppressing its activity. On the contrary, HSP90 inhibition induces the release of HSF1 from the HSP90:HSF1 complex, resulting in HSF1 activation and the transcription of several HSPs, including HSP70 [30].

A natural product from a bacterial source called Geldanamycin (GA) (Figure S1A), has been identified as an inhibitor of HSP90, as it binds to the N-terminal ATP-binding site and inhibits the ATPase activity of this chaperone [36]. GA, as well as its analogues, have shown neuroprotective effects against α-Syn aggregation in various cellular and animal models of PD. In the yeast *Saccharomyces cerevisiae* PD model, where the yeast expressed human wild-type α-Syn, and the A30P or A53T inherited mutant forms; Flower et al. found that GA treatment protected the cells against α-Syn stimulated apoptosis by reducing the accumulation of Reactive Oxygen Species (ROS). The authors also demonstrated that the overexpression of the yeast SSA3 (yeast HSP70) protected the cells in a similar manner. Collectively, Flower et al. speculated that the treatment with GA led to an increase in the activity of protective proteins, namely SSA3, which then inhibited the cascade of α-Syn toxicity events in yeast [37]. McLean et al. reported that an increase in the expression of HSP70 is positively correlated to the amounts of GA used to treat wild-type H4 neuroglioma cells and that there was a continual increase in the expression of HSP70 up to one day after GA treatment. The authors also observed that GA treatment before transfection of cells with both Synphilin-1 and Syn-T reduced the development of α-Syn inclusions and related neurotoxicity. However, GA treatment during and after transfection did not protect the cells from developing toxic inclusions [38]. In agreement, Emmanouilidou et al. reported the stimulatory effect of GA on HSP70 and the associated reduction of α-Syn soluble oligomers associated with proteasome fractions, as well as the Ubiquitin Proteasome System (UPS) restorative effect in PC12 cells overexpressing α-Syn A53T [39]. The positive effects of GA and the plausible mechanism of action have also been elucidated in the PD model of *Drosophila* flies [40] as well as in mouse [41] models, underlying the pivotal role of HSF1, the master transcriptional regulator of cellular response to proteotoxicity. In particular, in the 1-methyl-4-phenyl-1,2,3,6-tetrahydropyridine (MPTP) PD mouse model, Shen et al. showed that intracerebroventricular injection of GA (effective dose 10 μg/kg), 24 h before PD induction, resulted in reduced dopaminergic neurotoxicity. Trying to evaluate the mechanism behind this phenomenon, the authors observed a decrease in the level of HSP90, an increased expression of HSF1 and HSP70, as well as an escalated binding of HSF1 to HSP70 promoter region in the striatum of the mice [41].

Analogues of GA, such as 17-(Allylamino)-17-demethoxygeldanamycin (17-AAG) (Figure S1B) and 17-dimethylaminoethylamino-17-demethoxy-geldanamycin (17-DMAG) (Figure S1C), penetrate the Blood–Brain Barrier (BBB) more efficiently and are less toxic than GA [42]. These compounds are also able to bind to the ATP pocket of HSP90 in a GA-like manner to elicit similar response on HSPs. 17-AAG has been reported to reduce the aggregates and toxicity of α-Syn by interfering with HSP90 activities, in accordance with the mechanism whereby the inhibition of HSP90 triggers the activation of HSF1 and subsequent stimulation of HSP70/HSP40 to counteract the protein misfolding and/or aggregation events [43]. Unfortunately, despite the promising therapeutic effects of GA and some of its analogues in cellular and animal models of PD, pharmacokinetic issues such as hepatotoxicity and poor BBB permeability, which are likely to reduce the efficacy of these drugs in clinical scenarios, have led researchers to focus on finding pharmacological solutions through additional drug development and manipulation [44]. Xiong et al. developed a new GA derivative, a 19-phenyl-GA (a 19-substituted benzoquinone ansamycin), with improved redox stability and reduced toxicity in SH-SY5Y cells with respect to GA, as determined by apoptosis, oxygen consumption and 3-(4,5-dimethylthiazol-2-yl)-2,5-diphenyltetrazolium bromide assays. However, the authors reported that 19-phenyl-GA retained the beneficial properties of GA, including induction of HSP70 and HSP27, and reduction of α-Syn aggregation and related detrimental effects [44]. In a similar manner to this GA derivative, Putcha et al. investigated the α-Syn aggregation events and toxicity attenuation capacity of novel synthetic small molecules (SNXs) with GA-like HSP90 inhibitory activity, but with better oral bioavailability and magnified target specificity. Among the different SNXs tested with proven prevention of α-Syn aggregation of more than 75%, SNX-0723, SNX-3723, SNX-8891, and SNX-3113 were found to be the most effective in α-Syn wild-type transfected H4 cells; with an EC_50_ of 48 nM, SNX-0723 (Figure S1D) proved to be the best in terms of pharmacological properties. The authors also observed that SNX-0723 reached maximum brain concentrations 6 h after oral gavage administration of 10 mg/kg in rats and almost complete clearance after 24 h [43].

Just like GA, Radicicol, an antioxidant compound that is from a fungal source (Figure S1E), can bind to the N-terminal ATP-binding site of HSP90 with high specificity [36]. In an A53T PD yeast model, Derf et al. observed its ability to counteract α-Syn-induced cytotoxicity, inhibit the production of ROS, and offer protection against loss of mitochondrial membrane potential, nuclear DNA fragmentation, and apoptosis. The authors also reported its anti-apoptotic effect in mouse neuroblastoma Neuro2A cells transfected with wild-type α-Syn [45].

Just as small molecules that inhibit HSP90 activity have been shown to offer protection against α-Syn aggregates and related toxicity, other molecules that inhibit the activities of co-chaperones of HSP90 may have similar effects, emphasizing a possible mechanism independent of HSP90 inhibition. Co-chaperone p23 is one of the key components of the HSP90 machinery. Even though the exact mechanisms remain not completely elucidated, p23 has been shown to stabilize HSP90’s closed conformation and reduce its ATPase activity, thus playing an important role during client proteins maturation [46]. The small molecule Gedunin (Figure S1F), a naturally occurring pentacyclic triterpenoid extracted from *Azadirachta indica* and *Cedrela odorata*, is an inhibitor of p23 [47,48]. Gedunin has been reported to have a neuroprotective function against 1-methyl–4-phenyl-pyridinium (MPP^+^)-induced mitochondrial stress neurotoxicity in both rat dopaminergic N27 cells and human-induced Pluripotent Stem Cells (iPSC)-derived neurons [49]. Accordingly, Gedunin produces this effect by directly binding to p23, disrupting the interaction between the p23:HSP90 complex and Prolyl Hydroxylase Domain Protein 2 (PHD2), leading to the degradation of the PHD2 protein, and as a result, the activation of the transcription factor Hypoxia-Inducible Factor 1 α (HIF1 α) [49,50]. Based on this effect of Gedunin, it was not surprising that in different in vivo models of PD, Deoxygedunin, a natural derivative of Gedunin, was reported by Nie et al. to protect dopaminergic neurons through the activation of the TrkB signalling cascades [51].

Thus far, none of the first as well as the second generation (those with better pharmacokinetic properties) small-molecule HSP90 inhibitors (e.g., GA, Radicicol, and their analogues) have been successful in clinical trials [52]. However, their reported neuroprotection in cellular and animal models of PD by inhibiting HSP90, subsequently leading to the activation of HSP70/HSP40, as well as their effects on other pathways (e.g., autophagy, mTOR signalling pathways) could be a starting point for the development of other small molecules to counteract neurological disorders [53]. Thus, further efforts in finding more natural and/or synthetic HSP90 inhibitors, and/or to develop modified forms with better pharmacological capabilities, could greatly facilitate the search for the long-awaited drug to treat PD. Also, there should be further research to unravel the exact mechanism(s) of these HSP90 inhibitors against α-Syn aggregation and toxicity. For instance, the neuroprotective effect of 19-phenyl-GA might not only be due to its inhibitory effect on HSP90 but also to its reported ability to inactivate mTOR signalling pathways [44].

Interestingly, other small molecules can have a direct effect on HSF1 and/or HSP70 without recognized inhibition of HSP90. Carbenoxolone (Figure S1G) and U-133 are two examples of these small molecules. Their effects have been seen in both cellular and animal models. Carbenoxolone, a synthetic derivative of glycyrrhizic acid, prevents α-Syn aggregation-associated cytotoxicity by reducing these aggregates through the direct activation of HSF1 and increasing the expression of HSP70 in H4 cells overexpressing wild-type α-Syn [54]. In animal models, this compound was shown to offer neuroprotection against rotenone, an inhibitor of mitochondrial complex I. The treatment with Carbenoxolone (20 mg/kg by intraperitoneal injection for five weeks) reduced α-Syn aggregation and toxicity, led to the loss of dopaminergic neurons, and improved the observed motor dysfunction associated with the PD rat model. These effects were achieved through the activation of the HSF1-HSPs pathway [55].

Neurodegeneration can be halted, and associated behavioural disruption avoided through pharmacological stimulation of HSF1 and the increased expression of inducible HSP70 with U-133 (an acetylated tris-O-glucoside echinochrome), a derivative from the natural sea urchin pigment echinochrome [56]. This compound has been shown to be safe in both cellular and animal models [57]. Similar to the mechanism of Carbenoxolone discussed above, U-133 (5 mg/kg by intraperitoneal injection after lactacystin PD induction) has been shown to decrease α-Syn-associated neurodegeneration and neuroinflammation in both the preclinical [58] and clinical stage [56] of PD in rat.

**Table 1:** Summary of small molecules that target chaperone activity with their proposed mechanism and effects in different PD models.

Small Molecule	PD Model(s)	Target(s)	Main Proposed Effects after Small Molecule Administration	Reference
Geldanamycin	*Saccharomyces cerevisiae* expressing human wild-type, A30P or A53T α-Syn	SSA3	↓ oxidative stress ↓ apoptosis	[37]
	H4 cells co-transfected with Synphilin-1 and Syn-T	HSP70	↑ HSP70 ↓ α-Syn aggregation and related toxicity	[38]
	PC12 cells overexpressing A53T α-Syn	HSP70	↑ HSP70 ↓ α-Syn oligomers level ↑ UPS activity	[39]
	*Drosophila* flies expressing α-Syn	HSF1 HSP70	↑ HSP70 ↓ level of α-Syn associated toxicity	[40]
	Intraperitoneal MPTP treatment of male C57BL/6 mice	HSP90 HSF1 HSP70	↓ HSP90 ↑ HSF1 ↑ HSF1:HSP70 complex ↓ dopaminergic neurotoxicity	[41]
17-AAG	H4 cells transfected with wild-type α-Syn	HSP90 HSP70	↑ HSP70 ↓ α-Syn aggregates and cytotoxicity	[43]
19-phenyl-geldanamycin	SHSY5Y cells expressing A53T α-Syn	HSP70 HSP27	↑ HSP70 ↑ HSP27 ↑ cell viability ↓ higher-molecular-weight α-Syn oligomers ↑ early autophagic flux ↓ p-mTOR and p-p70S6K	[44]
SNX-0723	H4 cells transfected with wild-type α-Syn	HSP90 HSP70	↓ HSP90 ↑ HSP70 ↓ α-Syn oligomerization and cytotoxicity	[43]
Radicicol	Yeast expressing wild-type or A53T α-Syn; Neuro2A cells expressing wild-type α-Syn	HSP90	↓ cell death ↓ ROS levels ↓ loss MMP ↓ nuclear DNA fragmentation	[45]
Gedunin	MPP^+^ treatment N27 cells and iPSC-derived neurons	p23	↓ MPP^+^ induced neurotoxicity	[49]
Carbenoxolone	H4 cells overexpressing wild-type α-Syn	HSF1 HSP70	↑ HSF1 ↑ HSP70 ↓ α-Syn aggregates and cytotoxicity	[54]
	Subcutaneous injection of rotenone in male Sprague-Dawley rats	HSF1 HSPs	↑ HSF1 ↑ HSP70, HSP40, HSP27 ↓ α-Syn aggregation and ROS imbalance ↑ proteasome activity ↑ TH e dopamine levels ↑ motor functions	[55]
U-133	Microinjection of lactacystin in male Wistar rats	HSF1 HSP70 HSP40	↑ HSP70 ↓ dopaminergic neurons death ↑ TH and VMAT2 ↑ α-Syn aggregates ↓ microglial activation ↑ motor functions	[56]
	Intranasal administration of lactacystin in elderly male Wistar rats	HSF1 HSP70	↑ HSP70 ↓ activated microgliocytes ↓ α-Syn aggregates and phosphorylation ↓ neurodegenerative process	[58]

↑: Improvement/Increase; ↓: Reduction/Inhibition. 17-AAG: 17-(Allylamino)-17-demethoxygeldanamycin; MPTP: 1-methyl-4-phenyl-1,2,3,6-tetrahydropyridine; MPP^+^: 1-methyl–4-phenyl-pyridinium; iPSC: induced Pluripotent Stem Cells; HSP: Heat Shock Protein; HSF1: Heat Shock transcription Factor 1; UPS: Ubiquitin Proteasome System; ROS: Reactive Oxygen Species; MMP: Mitochondrial Membrane Potential; TH: Tyrosine Hydroxylase; VMAT2: Vesicular Monoamine Transporter 2.

HSP70 is one of the most studied molecular chaperones as a target of small molecules. However, one of the most interesting aspects yet to be clarified is the selectivity of these compounds, since several HSP70 members are differently expressed and localized in cells [59]. So, it would be very fascinating to highlight the interaction between small molecules and a particular HSP70 member, in contributing to the acquisition of adequate treatment for PD.

Overall, the data discussed above underlie the positive effects exerted by both natural and synthetic small molecules in counteracting the burden of α-Syn aggregates and cytotoxicity, thanks to their ability to inhibit HSP90 activity and/or upregulate, mainly, HSP70. However, we underline some critical points. In-depth research are needed to better elucidate the possible interaction of these compounds with other key proteins, in order to avoid the modulation of other relevant signalling pathways. Keeping in mind that the inhibition of one molecular chaperone can result in the upregulation of other HSPs and vice versa, it would be interesting to explore the effects of the administration of these small molecules on different HSP family members, thus highlighting a possible synergic effect. Of note, the α-Syn aggregation pathway is extremely dynamic in the cells, meaning that different α-Syn forms could coexist (e.g., monomers, oligomers, and fibrils) and each HSP can exert its function on a specific form. We also evaluated that most of the research have been carried out in cellular models of PD. Thus, many efforts are needed to move from cellular to animal models, and to translate the positive effects in clinical settings. The lack of clinical trials could also be as a result of inadequate data related to the safety and efficacy of these small molecules in in vivo models, issues with patient enrolments, and lack of funds [60].

## 3. Natural and Synthetic Small Molecules That Modulate the α-Syn Aggregation Pathway and Related Toxicity

As previously reported, some small molecules are able to impact the functions of certain chaperones in attenuating α-Syn aggregation and related pathology; however, others can interfere with different steps of the α-Syn aggregation process, exhibiting a “chaperone-like” activity. Thus, some small molecules can affect different α-Syn species prone to aggregation and/or the number of aggregates through various known and still unknown mechanisms. These molecules can be of plant, animal, and/or synthetic origin. Among them, Baicalein, Epigallocatechin-3-gallate (EGCG), Squalamine (SQU), and Trodusquemine (TRO) of natural origin, as well as molecular tweezer CLR01 of synthetic origin will be discussed (Table 2).

Baicalein (Figure S1H), a flavone with antioxidant properties, is the main component of the flowering plant *Scutellaria baicalensis*. This plant is distributed mainly across China, Korea, and Russia [61]. Zhu et al. have shown through in vitro experimental models that a low concentration of Baicalein, especially its quinone oxidized form, binds covalently to α-Syn oligomers through the formation of an imine Schiff base. According to them, this reaction stabilizes the α-Syn oligomeric form and inhibits fibril formation. The authors also observed that Baicalein caused the disaggregation of α-Syn fibrils to mainly soluble oligomeric forms [62]. These features of Baicalein were also confirmed in other in vitro studies [63,64,65] and in different cell lines and animal models of PD [64,65,66,67,68]. Accordingly, in cellular lines expressing wild-type or mutant forms of α-Syn, the treatment with Baicalein resulted in the reduction of α-Syn toxic aggregates [64,65,66]. Particularly, Li et al. observed that Baicalein improved the viability of wild-type α-Syn and A53T α-Syn transfected SN4741 dopaminergic cells, up to 12 h of treatment. Importantly, the authors also reported that Baicalein treatment increased macroautophagy, an effect that could be correlated to the reduction of α-Syn propagation between neighbouring cells [66]. In an in vivo PD mouse model, Hu et al. demonstrated Baicalein’s ability (100 mg/kg by intraperitoneal injection) to reduce the number of α-Syn oligomers in the midbrain, thoracic, spinal cord, and ileum. Using the grid and rotarod tests, the authors also reported that the motor impairments mediated by rotenone administration were significantly reduced with Baicalein treatment [67]. Fascinatingly, the administration of this small molecule showed no sign of toxicity in the kidney and liver functions of the mice [69]. In an MPP^+^ rat model of PD, Hung et al. also demonstrated that Baicalein has an anti-α-Syn aggregation as well as neuroprotective effects after 2 days of daily intraperitoneal treatment (effective dose 30 mg/kg) [68]. Among the several well-known effects (e.g., anti-inflammatory, anti-oxidative, and anti-apoptotic) of Baicalein, its anti-α-Syn aggregation function represents a recent line of research in PD [70].

Epigallocatechin-3-gallate (EGCG) (Figure S1I) is the most active polyphenol and has received the most attention among catechins found in green tea and represents between 200 and 300 milligrams (50% to 80%) per cup of brewed green tea [71]. EGCG is among the category of small molecule compounds with low toxicity, ease of availability, and permeability of the BBB to have attracted massive interest in their inhibitory role against the α-Syn fibrillization process and/or mature fibril destruction ability [72]. In vitro, EGCG has been shown to decrease α-Syn aggregation and toxicity by triggering several mechanisms [73,74,75,76,77]. For instance, using biophysical and biochemical techniques such as Thioflavin T (ThT) and Nitroblue Tetrazolium (NBT) staining binding assay, EGCG was observed to bind to monomers, preventing the α-Syn amyloidogenesis process and diverting the aggregation of monomeric α-Syn into non-toxic unstructured oligomers lacking the typical seeding capacity [73] or facilitating the formation of fibrils [74]. EGCG was also shown to attenuate the α-Syn aggregation process and related detrimental effects by forming EGCG/Cu(II) complex in α-Syn transduced-PC12 cells [75]. Moreover, EGCG was found to directly bind to the β-sheet-rich amyloid species, remodelling their structure, and causing the appearance of smaller disordered non-toxic protein aggregates in Pre-Formed Fibril (PFF)-treated HEK-293 and PC12 α-Syn transfected cells [73,76]. Another mechanism involves the capacity of EGCG to remodel the C-terminal of oligomers, resulting in a weakened interaction with the cell membrane, thus inhibiting the ability of α-Syn oligomers to generate cytotoxicity in OLN-93 rat brain cell line [78]. Of note, the α-Syn aggregation pathway has been demonstrated to significantly increase when α-Syn binds to lipid membranes [79]. However, regarding the pattern of interaction between α-Syn and EGCG, Nuclear Magnetic Resonance (NMR) spectroscopy revealed that EGCG has the highest affinity for the N-terminal region of α-Syn, although it can bind to all other regions [80]. In another study, Yang et al. observed that EGCG inhibits protofibril–membrane interplay by forming strong H-bonds and cation-π interactions with cellular membranes [81]. These pieces of evidence show that EGCG could be a therapeutic option in PD and other synucleinopathies, albeit the effect(s) of EGCG on the α-Syn aggregation pathway in vivo need to be explored more extensively.

Squalamine (SQU) (Figure S1J) is an organic compound discovered in 1993 from the stomach extracts of *Squalus acanthus*, the dogfish shark, and belongs to the class known as “Monohydroxy bile acids, alcohols, and derivatives” [82]. SQU exhibits different activities, such as anti-viral [83], anti-microbial [84], and anti-angiogenic [85] activity. Using NMR spectroscopy and ThT techniques, Perni et al. demonstrated that SQU directly interacts with α-Syn only when it is in high concentrations. Furthermore, the authors observed that SQU’s anti-α-Syn aggregation pathway is through its ability to competitively bind lipid membranes, displacing already bound α-Syn and/or preventing the binding of α-Syn to the membranes. They also demonstrated the beneficial effects of SQU administration in SHSY5Y cells, evidenced by the inhibition of α-Syn aggregation and related toxicity. Interestingly, similar inhibitory activity was observed in *C. elegans*, which has been genetically engineered to overexpress α-Syn. Also, there were reported improvements in motility, swimming speed, and paralysis rate [86], which will probably improve survival in the worms. These observations could be due to the high affinity of SQU to anionic phospholipids as a result of its net positive charge [83]. The effects of SQU in the restoration of PD-related gastrointestinal tract impairments have also been reported in mice [87]. The positive effects of SQU did not go unnoticed as this aminosterol compound finally entered clinical studies to target α-Syn aggregation in the Enteric Nervous System (ENS). Pharmacologically targeting the ENS might be because of the shreds of evidence which suggest that the ENS could be the initiation site of α-Syn aggregation, and then its subsequent transfer in a ‘‘prion-like fashion’’ to the CNS in PD [88]. In two human clinical trials (NCT03047629 and NCT03781791), an orally administered SQU phosphate (ENT-01) compound successfully improved gut dysmotility [89,90], and enhanced cognitive ability [90]. The only side effects reported were nausea and diarrhoea. Lengthier treatment periods need to be undertaken in future investigations to confirm its safety and tolerability, as well as to explore its effects against other cardinal motor and non-motor symptoms of PD.

Trodusquemine (TRO) (Figure S1K) is another naturally occurring aminosterol, discovered in 2001 from the liver of a dogfish shark and it is closely related to SQU. Specifically, TRO contains a spermine moiety instead of spermidine in the side chain [91,92]. Perni et al. observed that TRO exhibited similar effects against primary α-Syn nucleation events as reported about SQU above. Nevertheless, TRO inhibited both lipid-influenced primary nucleation as well as fibril-dependent secondary aggregation pathways, by displacing proteins from lipid membranes and directly binding to α-Syn fibrils, thus preventing its autocatalytic amplification. Interestingly, the TRO concentration needed to displace oligomers, the pathogenic forms of α-Syn, was lower than that needed to displace monomers [93]. With the reported physiological functions of membrane-bound α-Syn monomers [94], the selective displacement of oligomers could be a pharmacological step in the right direction. The authors also reported the beneficial effects induced by TRO administration, both in SH-SY5Y cells treated with α-Syn oligomers and in the *C. elegans* PD model [93]. Unlike SQU, which has entered clinical trials against PD, to the best of our knowledge, there are no ongoing clinical trials regarding TRO at the moment, even though its activity has been explored in other pathologies (e.g., cancer and type 2 diabetes). These SQU and TRO effects against the α-Syn aggregation pathway and related toxicity could just be a starting point to discover new therapeutic approaches in PD.

CLR01 (Figure S1L) is one of the leading molecular tweezers which has been found to have a similar activity to EGCG and is unsurprisingly gaining a lot of attention for its ability to inhibit the aggregation of several pathological proteins in different models [95]. CLR01 binds primarily to lysine residues. Thus, it disrupts both electrostatic and hydrophobic interactions needed for aggregation [96]. In vitro, CLR01 has been shown to arrest fibril formation and disaggregate PFF into non-fibrillar aggregates after it was added at different time points, as observed by Electron Microscopy (EM) analysis and ThT assay [96,97]. Bengoa-Vergniory et al. also demonstrated that CLR01 prevented recombinant α-Syn from forming fibrils and oligomers, and showed its anti-aggregation effect on LB extracts from the *Substantia Nigra pars compacta* (SNpc) of PD patients [97]. Acharya et al. argued that the anti-aggregation activity of CLR01 is a result of its predominant binding to the N-terminal Lys-10/Lys-12, leading to an increased reconfiguration rate of the aggregates, hence interrupting the interaction necessary for the aggregation process [98]. In HEK 293 cells expressing wild-type human α-Syn and in differentiated PC12 cells treated with α-Syn oligomers, CLR01 inhibited α-Syn toxicity, decreasing cell death without affecting the proliferation rate [96]. Furthermore, CLR01 efficiently reduced aggregation and related toxic effects in α-Syn-transfected SHSY5Y, in iPSC-derived dopaminergic neurons, and in primary rat cortical cultures treated with LB extracts from PD patients [97]. In an in vivo Zebrafish (ZF) model of PD, CLR01 was found to ameliorate the neurotoxicity induced by the administration of the pesticide Ziram [99]. Earlier, Prabhudesai et al. reported the rescue effect of CLR01, in terms of embryo survival, reduction of α-Syn aggregates and apoptosis, and the restoration of UPS activity in ZF-expressing human wild-type α-Syn [96]. In a PD mouse model overexpressing human α-Syn, which mimics the human pathology (SNCA-OVX), the 2-month CLR01 treatment (40 μg/kg/day by subcutaneous injection) of 12-month-old mice was seen to suppress oligomeric accumulation and successfully improved motor behaviour. Even in the case of 18-month-old mice treated for one month, which probably would have lost about one-fourth of their dopaminergic neurons, CLR01 (40 μg/kg/day by osmotic mini-pump) effectively lowered the number of α-Syn oligomers in SN neurons, astrocytes, and microglia, as well as increased levels of Peroxisome Proliferator Gamma Co-activator 1 (PGC1α) and Lysosome-associated membrane protein 2 (Lamp2A). However, the treatment was unable to restore motor deficits. In the same study, one-month CLR01 treatment also decreased α-Syn aggregation after intrastriatal injection of recombinant α-Syn PFF or injection of LB extracts obtained from PD patients into the SN of 3-month-old C57BL/6 wild-type mice (400 μg/kg/day CLR01 subcutaneous injection) and 4-month-old C57BL/6 wild-type mice (CLR01 40 μg/kg/day by osmotic mini-pump), respectively [97].

**Table 2:** Summary of small molecules that modulate the α-Syn aggregation pathway with their proposed mechanism and effect in different cell lines and animal models of PD model.

Small Molecule	PD Model(s)	Main Proposed Effects after Small Molecule Administration	Reference(s)
Baicalein	Differentiated PC12 cells and N2A cells expressing E46K α-Syn	↓ cell death ↓ mitochondrial dysfunction ↓ proteasome inhibition ↓ α-Syn aggregation	[64]
	SN4741 cells expressing wild type or A53T α-Syn	↑ cell viability ↑ macroautophagy ↓ secretion of α-Syn species	[66]
	Intragastric rotenone administration in male C57BL/6 mice	↓ α-Syn aggregation ↑ striatal neurotransmitters concentration ↑ behavioural functions	[67]
	Intranigral infusion of MPP^+^ in male Sprague-Dawley rats	↓ α-Syn aggregation ↓ loss of nigrostriatal dopamine content ↓ neuroinflammation ↓ MPP^+^-induced apoptosis and autophagy	[68]
EGCG	PC12 cells overexpressing wild-type α-Syn	↓ α-Syn protein overexpression and aggregation ↓ Cu(II)-induced toxic conformation transition of α-Syn, by forming Cu(II)/EGCG complex ↓ Cu(II)-induced α-Syn protein overexpression and aggregation ↓ Cu(II)-induced cytotoxicity	[75]
	HEK-293 cells overexpressing wild-type α-Syn treated with PFF; PC12 cells treated with PFF	↓ NP-40 fraction of α-Syn aggregates ↑ SDS-stable fraction of α-Syn aggregates ↓ α-Syn aggregates cytotoxicity	[73,76]
	OLN-93 cells treated with α-Syn oligomers	↓ α-Syn oligomers cytotoxicity	[78]
Squalamine	SH-SY5Y cells treated with α-Syn oligomers	↓ α-Syn oligomers cytotoxicity ↓ ROS production ↓ α-Syn oligomers bound to plasma membrane	[86]
	*Caenorhabditis elegans* overexpressing α-Syn	↓ α-Syn inclusions ↑ worms motility	
	Mice overexpressing A53T human α-Syn	↑ propagating contractile clusters velocity in vitro ↑ colonic motility in vivo ↑ ENS function	[87]
Trodusquemine	SH-SY5Y cells treated with α-Syn oligomers	↓ α-Syn oligomer cytotoxicity ↓ ROS production ↓ α-Syn oligomers bound to plasma membrane	[93]
	*Caenorhabditis elegans* overexpressing α-Syn	↓ α-Syn inclusions ↑ worm motility ↑ worm longevity	
CLR01	HEK 293 cells expressing wild-type human α-Syn; differentiated PC12 treated with α-Syn oligomers	↓ cell death	[96]
	Zebrafish expressing human wild-type α-Syn	↑ embryon survival and normal phenotype ↓ α-Syn aggregates/clumps ↓ apoptosis ↑ UPS activity	
	SH-SY5Y cells overexpressing α-Syn	↓ α-Syn aggregates	[97]
	iPSC-derived dopaminergic and primary rat neuronal cultures treated with LB extracts	↓ α-Syn aggregates and cytotoxicity ↓ axonal degeneration ↓ interaction of α-Syn oligomers with axonal transport proteins	
	Transgenic SNCA-OVX mice (12 months old)	↓ α-Syn oligomers and astrogliosis ↑ motor function	
	C57BL/6 wild-type mice injected with mouse PFF in the dorsal striatum or LB fractions in SN	↓ α-Syn aggregation	

↑: Improvement/Increase; ↓: Reduction/Inhibition. EGCG: Epigallocatechin-3-gallate; MPP+: 1-methyl–4-phenyl-pyridinium; PFF: Pre-Formed Fibril; iPSC: induced Pluripotent Stem Cells; LB: Lewy Body; SN: Substantia Nigra; ROS: Reactive Oxygen Species; ENS: Enteric Nervous System; UPS: Ubiquitin Proteasome System.

As previously discussed regarding small molecules that affect the activity of molecular chaperones, despite the encouraging results obtained by the administration of small molecules that directly interact with α-Syn, some critical points need special attention. Particularly in this context, the dynamic process of α-Syn aggregation plays a pivotal role, therefore the simultaneous presence of different α-Syn forms can reduce the protective effect of these compounds. Specifically, the capacity of the small molecules to interfere with the aggregation process is strictly related to the biophysical properties of the α-Syn structures acquired in different conditions, e.g., unfolded monomeric form in an aqueous environment, α-helix structure when it interacts with lipid membranes, and β-sheet regions in the oligomeric form. Also, the post-translational modification (e.g., phosphorylation, lipidation, and SUMOylation) can modulate the capacity of the small molecules to counteract the α-Syn-aggregation process [100]. Accordingly, extensive research are needed in the future to tackle this issue.

## 4. Conclusions

PD is the second most common brain progressive disorder, characterized by cardinal motor and non-motor symptoms. Currently, the therapies for PD patients aim to slow down and counteract the signs of manifestation because disease-modifying approaches are not yet available. Although the pathophysiological mechanisms underlying PD, as well as other protein-misfolding diseases, have still not yet been fully elucidated, the common features of these disorders involve the gain of toxic structural conformation(s) of key proteins and the disruption of different branches of PN. Specifically in PD progression, α-Syn loses its native structure and acquires different misfolded forms that generate LBs that can be identified both in CNS and in ENS. Accordingly, understanding the mechanisms that drive the multistep α-Syn aggregation pathway emerges as one of the major research fields aiming to develop successful strategies to inhibit α-Syn aggregation events and/or the processes that may lead to the spreading of aggregates between neighbouring cells. These have led to an increase in the literature data that shed light on the role exerted by natural and/or synthetic small molecules in modifying the activities of molecular chaperones, as master regulators of proteostasis. Moreover, there is an increasing attention paid to small molecules that can interfere directly with the α-Syn-aggregation events, thus exerting a “chaperon-like” activity. The increased interest in this area of research is due to certain advantages of these compounds, such as bioavailability, stability, ability to cross cell membranes, and feasibility of oral administration [101].

Globally, the results provided by the scientific literature highlight the beneficial effects of the administration of both natural and synthetic small molecules to manage the α-Syn-aggregation process and its associated neurotoxic effects. These compounds can exert their activity inside the cells to maintain and/or restore the proteome balance (mainly inhibiting HSP90 activity and/or promoting HSP70 activity), but can also interfere directly with different toxic forms of α-Syn to reduce the number and the dimension of the aggregates. Both strategies reduce the detrimental effects associated with α-Syn aggregation events and modulate the activity of different signalling pathways (e.g., TrkB, mTOR, and HIF1a), macroautophagy, and mitochondrial activity. Of note, very interesting results are related to the ability of small molecules such as EGCG, SQU, and TRO to interfere with α-Syn species and lipid membranes, a type of activity previously associated with molecular chaperones such as HSP70, HSP60, HSP90, and small HSPs, thus highlighting a possible “lipid chaperon-like” activity of these small molecules [102,103]. This feature is of specific importance for PD pathophysiological mechanisms, taking into account not only the clearance of preformed α-Syn aggregates via autophagy lysosomal pathway but also the spreading of the aggregates in different areas of the brain and between organs (i.e., “gut-brain axis” communication pathway).

Although the data obtained until now on the possible use of natural and synthetic small molecules as therapeutic strategies for PD and other synucleinopathies are very engaging from a critical point of view, further studies are needed to better elucidate the role exerted by these compounds on other signalling pathways, as well as their selectivity and binding mechanism to the different forms of α-Syn. Moreover, we underline that most of the studies have been performed in in vitro and animal models with only a few compounds being studied in clinical trials. Although animal models are crucial in improving the knowledge about the molecular pathways triggered in different diseases, it is not possible to directly extend the obtained results to cells, tissues, and the whole human body. Moreover, more data are needed in terms of pharmacokinetic parameters of the small molecules, such as absorption, BBB permeability, hepatotoxicity, and excretion. With regard to future developments, one of the intriguing experimental approaches could be the co-administration of small molecules together with different mechanisms of action, such as the modulation of chaperone activity, direct interaction with different α-Syn forms, and those that can improve proteasomal degradation. This approach might trigger a synergic effect and reduce the concentration of these compounds needed to obtain positive effects, as well as to reduce their possible side effects. Another fascinating challenge could be the combination of different therapeutic strategies, such as the administration of small molecules with synthetic peptides, antibodies, or other biologic drugs to counteract α-Syn burden. In conclusion, the improvement in this area of research could result in the design and/or development of novel drugs able to modify the progression of PD.

## Data Availability

Not applicable.

## Supplementary Materials

The following supporting information can be downloaded at https://www.mdpi.com/article/10.3390/ijms241713370/s1.
