# Supplementary material for: Uncovering the Role of Natural and Synthetic Small Molecules in Counteracting the Burden of α-Synuclein Aggregates and Related Toxicity in Different Models of Parkinson’s Disease
Source: Int J Mol Sci. 2023 Aug 29;24(17):13370. doi: 10.3390/ijms241713370 (PMC10488152; doi:10.3390/ijms241713370)
Supplement: Supplementary file 1 [file ijms-24-13370-s001.zip › ijms-2566197-supplementary.pdf]

Figure S1. Molecular structure, source, molecular formula, and molecular weight of the discussed small molecules.

A. Geldanamycin

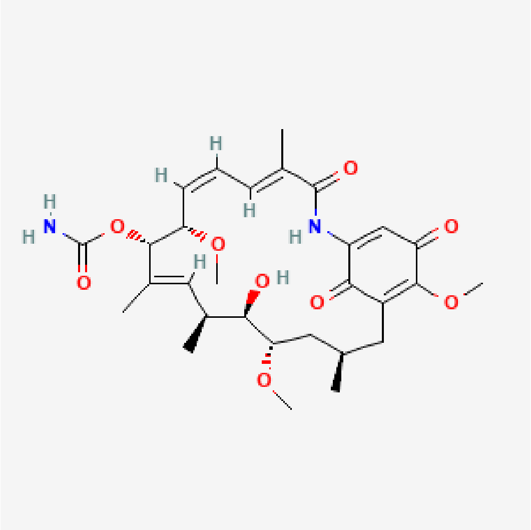

PubChem CID: 5288382
Source: Bacteria (*S. hygroscopicus*)
Molecular Formula: $C_{29}H_{40}N_2O_9$
Molecular Weight: 560.6 g/mol

B. 17-AAG

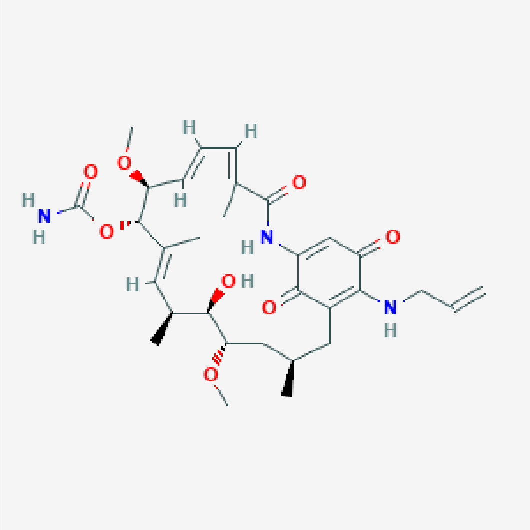

PubChem CID: 6440175
Source: Semi-synthetic derivative of GA
Molecular Formula: $C_{31}H_{43}N_3O_8$
Molecular Weight: 585.7 g/mol

C. 17-DMAG

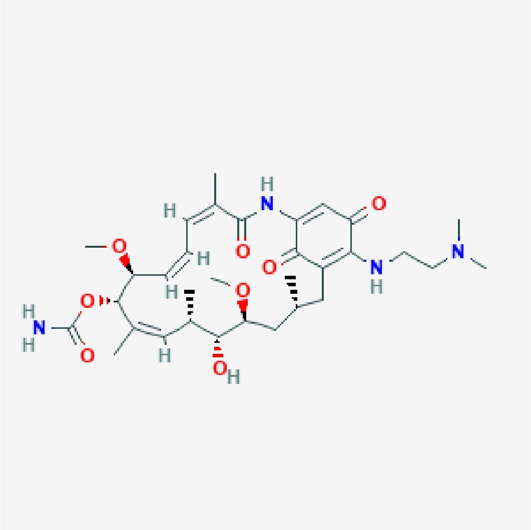

PubChem CID 9852573
Source: Semi-synthetic derivative of GA
Molecular Formula: $C_{32}H_{49}ClN_4O_8$
Molecular Weight: 653.2 g/mol

D. SNX-0723

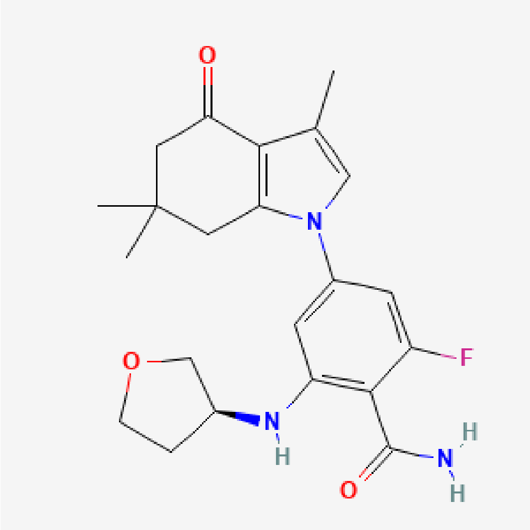

PubChem CID 67495834
Source: Synthetic
Molecular Formula: $C_{22}H_{26}FN_3O_3$
Molecular Weight: 399.5 g/mol

E. Radicicol

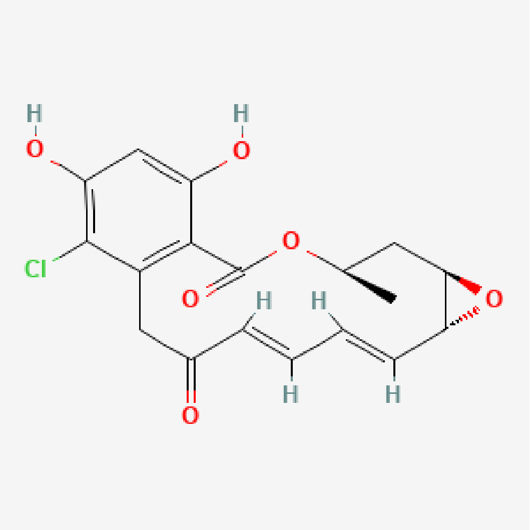

PubChem CID: 6323491
Source: *N. quadriseptata, H. fuscoatra, M. bonorden* and other fungi
Molecular Formula: $C_{18}H_{17}ClO_6$
Molecular Weight: 364.8 g/mol

F. Gedunin

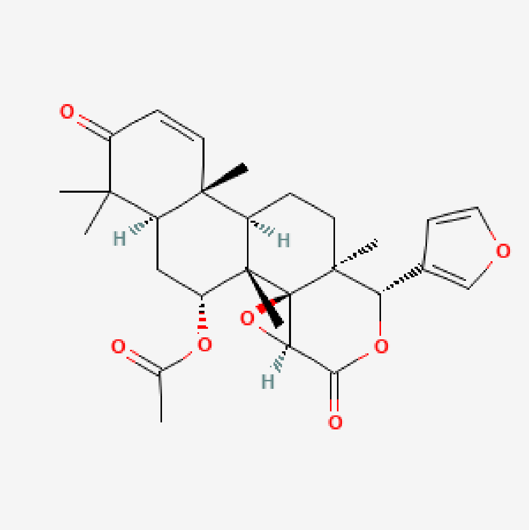

PubChem CID: 12004512
Source: *A. indica, C. odorata*
Molecular Formula: $C_{28}H_{34}O_7$
Molecular Weight: 482.6 g/mol

G. Carbenoxolone

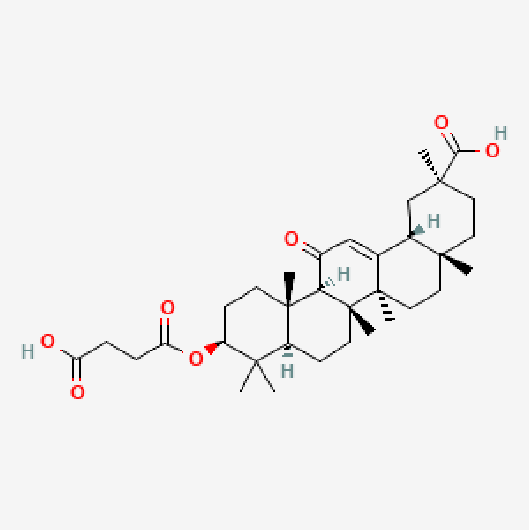

PubChem CID: 636403
Source: Synthetic
Molecular Formula: $C_{34}H_{50}O_7$
Molecular Weight: 570.8 g/mol

H. Baicalein

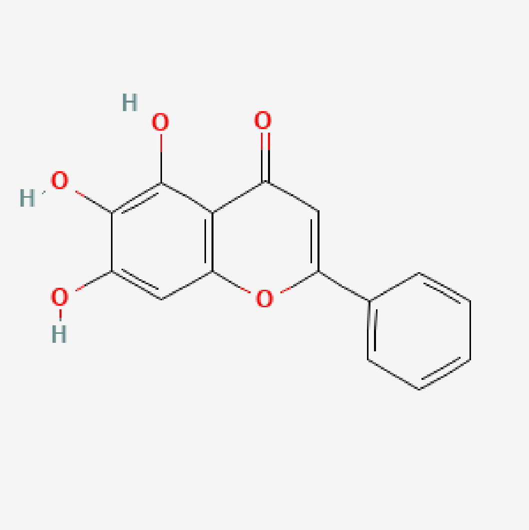

PubChem CID: 5281605
Source: *Scutellaria baicalensis* (Plant)
Molecular Formula: $C_{15}H_{10}O_5$
Molecular Weight: 270.24 g/mol

I. EGCG

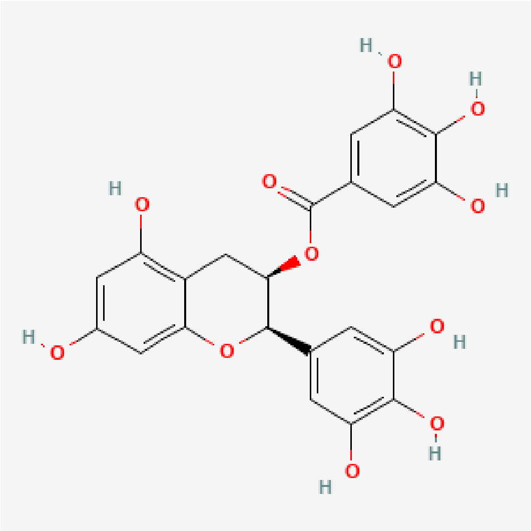

PubChem CID: 65064
Source: Green Tea
Molecular Formula: $C_{22}H_{18}O_{11}$
Molecular Weight: 458.4 g/mol

J. Squalamine

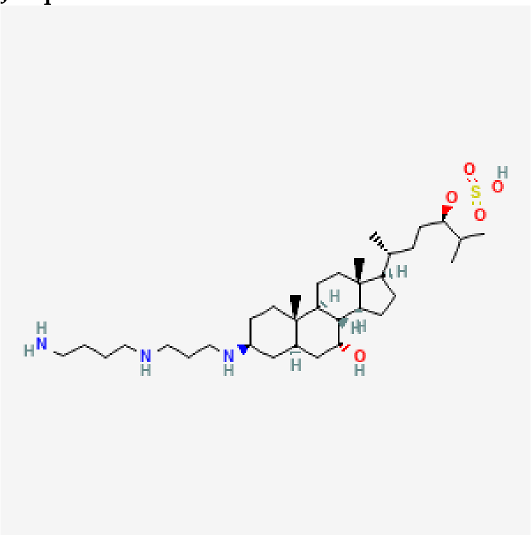

PubChem CID 72495
Source: *Squalus acanthias*
Molecular Formula: $C_{34}H_{65}N_3O_5S$
Molecular Weight: 628 g/mol

K. Trodusquemine

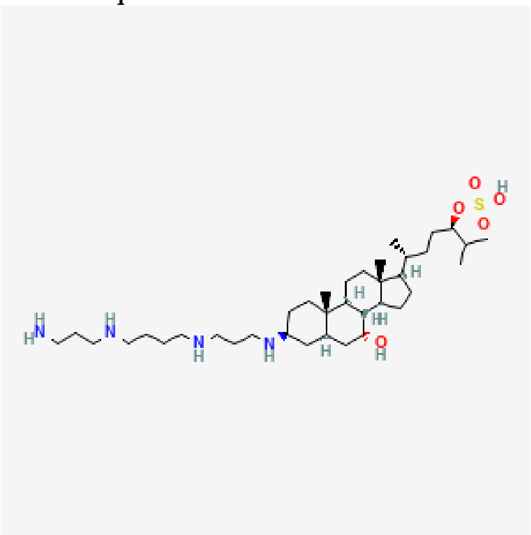

PubChem CID: 9917968
Source: *Squalus acanthias*
Molecular Formula: $C_{37}H_{72}N_4O_5S$
Molecular Weight: 685.1 g/mol

L. CLR01

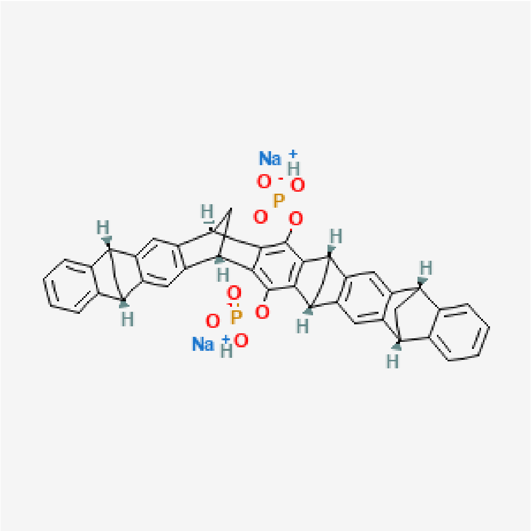

PubChem CID: 54758480
Source: Synthetic
Molecular Formula: $C_{42}H_{30}Na_2O_8P_2$
Molecular Weight: 770.6 g/mol

All the chemical structures, molecular formula, and molecular weight were adapted from Pubchem, National Library of Medicine (https://www.ncbi.nlm.nih.gov/). 17-AAG: 17-(Allylamino)-17-demethoxygeldanamycin; 17-DMAG: 17-dimethylaminoethylamino-17-demethoxy-geldanamycin; EGCG: Epigallocatechin-3-gallate.
